# The chain mediating roles of stigma and health-promoting lifestyle in the relationship between disability index and posttraumatic growth among patients with inflammatory bowel disease

**DOI:** 10.3389/fpubh.2026.1816875

**Published:** 2026-07-10

**Authors:** Yi Wang, Yamei Chen, Xinyi Gao, Linjuan Xun, Baixue Jiang, Xiaoping Jin

**Affiliations:** Department of Nursing, Shanghai Tenth People’s Hospital, Shanghai, China

**Keywords:** disability index, health-promoting lifestyle, inflammatory bowel disease, posttraumatic growth, stigma

## Abstract

**Objective:**

To explore the levels of disability, Health-Promoting Lifestyle Profile II (HPLP-II), illness-related stigma, and posttraumatic growth (PTG) in patients with inflammatory bowel disease (IBD), and to examine the chain mediating roles of HPLP-II and illness-related stigma in the association between disability and PTG.

**Methods:**

A cross-sectional survey was performed among 311 IBD patients recruited from three tertiary hospitals in Shanghai between August 2022 and April 2023. All participants completed standardized questionnaires, including general information scale, IBD Disability Index (IBD-DI), PTG Inventory (PTGI), HPLP-II and Stigma Scale for Chronic Illness (SSCI). SPSS 26.0 and Hayes’ PROCESS macro (Model 6, 5,000 bootstrap resamples) were adopted for data analysis. Disease activity and disability severity were set as covariates in the mediation model.

**Results:**

The mean scores of PTGI, IBD-DI, HPLP-II and SSCI were 70.84 ± 25.65, 28.39 ± 12.98, 148.61 ± 36.84 and 69.24 ± 29.33, respectively. IBD-DI was negatively correlated with HPLP-II and PTGI, and positively correlated with SSCI; SSCI was negatively correlated with HPLP-II and PTGI, while HPLP-II was positively correlated with PTGI (all *p* < 0.001). Chain mediation analysis showed no significant direct effect between IBD-DI and PTG (*β* = 0.042, *p* = 0.539). HPLP-II and illness-related stigma exerted independent and chain mediating effects in this association. The total indirect effect was −1.019. The chain mediation pathway, independent pathway of HPLP-II and illness-related stigma accounted for 25.87%, 64.71%, and 9.42% of the total association, respectively.

**Conclusion:**

Disability is not directly correlated with PTG in IBD patients. Its negative linkage with PTG is mediated by the chain pathway of impaired health-promoting lifestyle and elevated illness-related stigma. Targeted interventions to reduce stigma and optimize health-promoting lifestyle may help improve PTG and promote psychological rehabilitation among IBD patients.

## Introduction

Inflammatory bowel disease (IBD), primarily comprising ulcerative colitis (UC) and Crohn’s disease (CD), is a chronic, relapsing inflammatory disorder of the gastrointestinal tract (1, 2). Data from the Global Burden of Disease study indicate that while the incidence of IBD has stabilized in Western developed countries, it is rising rapidly in newly industrialized nations, a trend paralleling socioeconomic transitions and changes in modern lifestyle (3). Large-scale epidemiological data from China report an incidence of 10.04 per 100,000 population, establishing IBD as a significant national public health concern (4). Beyond enduring recurrent gastrointestinal symptoms and substantial healthcare burdens, patients with IBD frequently experience persistent, multidimensional impairments in physical health, psychological well-being, and social functioning, which severely compromises their overall quality of life (5–7).

Within the framework of the World Health Organization’s International Classification of Functioning, Disability and Health (ICF), IBD-related disability extends beyond organic pathology to encompass comprehensive functional impairments, including physiological dysfunction, activity limitations, and restrictions in social participation (8, 9). Chronic intestinal inflammation, which is accompanied by persistent mucosal injury and impaired repair, constitutes the primary pathological basis for this disability (10). Meta-analytic evidence reveals that the global pooled prevalence of moderate-to-severe disability in IBD is 29.6%, escalating to 56.9% during active disease and persisting at 27.0% even during clinical remission (11). Data from Chinese cohorts indicate that over 24.4% of IBD patients experience varying levels of functional disability (12). The chronic and progressive nature of this disability is closely linked to long-term clinical outcomes and the efficacy of individualized rehabilitation, posing a sustained burden to patients and healthcare systems worldwide (13).

Notably, despite the dual stresses of disease activity and functional disability, a subset of patients with IBD achieves posttraumatic growth (PTG) through active psychological adjustment (14, 15). PTG describes positive psychological changes in self-perception, interpersonal relationships, and philosophy of life that can emerge following a struggle with severe illness or other highly challenging life events. It is observed alongside cognitive restructuring and emotional regulation, reflecting an individual’s psychological resilience and adaptive potential (16). In the context of IBD, typical PTG outcomes include greater disease acceptance, improved self-management, and a re-evaluated sense of life priorities (17). As a critical behavioral foundation, the adoption of health-promoting lifestyle (HPL) can strengthen disease-related self-efficacy and improve long-term disease control, and is correlated with the development of PTG in this population (18). For patients facing a lifelong condition, PTG represents a key psychological marker of favorable long-term adaptation; however, its underlying associative pathways require further clarification.

Health-promoting lifestyle (HPL) serves as an essential set of behavioral strategies for patients to manage IBD, maintain well-being, and mitigate disease-related stress (19). Grounded in Pender’s Health Promotion Model, HPL encompasses multiple dimensions, including nutrition, physical activity, stress management, interpersonal support, and health responsibility. In the context of IBD, HPL extends beyond general wellness behaviors and is specifically operationalized as a set of disease-tailored actions, such as following dietary modifications, maintaining adequate physical activity within tolerable limits, practicing effective stress management, and demonstrating consistent medication adherence (20). Research, such as that by Walker et al., suggests that HPL can improve self-efficacy, ameliorate physical symptoms, and reduce psychological distress, and is associated with positive psychological adaptation and bridging physical and mental health (21). Positive correlations between HPL and PTG have been documented; these health-promoting behaviors are associated with reduced stress-induced negative emotions, which correspond to favorable behavioral and psychological preconditions for PTG (22). Specifically, regular exercise, balanced nutrition, and adequate sleep are associated with modulation of intestinal inflammation and emotional distress via the gut-brain axis, and correspond to the alleviation of anxiety and depression and the optimization of long-term psychosomatic prognosis. Collectively, these behaviors constitute a core, lifelong holistic management strategy for IBD (18).

However, the potential for positive psychological growth is often undermined by illness-related stigma. As a prevalent negative psychosocial burden in IBD, stigma arises from the disease’s unique intestinal symptoms, treatment features, and societal stereotypes, presenting as perceived discrimination and social devaluation (23, 24). In IBD, the experience of stigma is often rooted in the specific, sometimes invisible, yet disruptive symptoms of the disease, such as unpredictable bowel urgency, fears of fecal incontinence, and the social embarrassment tied to ostomy management (23). The stigma is reinforced by societal taboos surrounding bowel symptoms, which are associated with self-devaluation, social withdrawal, and impaired help-seeking behavior (25, 26). This experience is associated with adverse psychological and behavioral patterns, including diminished self-esteem and social avoidance. Multiple studies confirm that a higher degree of functional disability in IBD is significantly associated with a greater perception of illness stigma (27, 28). Furthermore, intense stigma can undermine patients’ adherence to healthy behaviors, while attempts to conceal the illness may paradoxically exacerbate interpersonal difficulties (26). The relationship between HPL and stigma is dynamic and bidirectional. On the one hand, stigma can discourage individuals from engaging in HPL activities, such as exercising in public or participating in group activities, due to fear of symptom exposure. On the other hand, a lower engagement in HPL—which may be associated with functional limitations—can amplify feelings of illness-related stigma, as patients perceive themselves as less capable of performing health-promoting activities, and is associated with a sense of otherness (29). Therefore, exploring the potential mediating role of stigma in the relationship between disability and PTG provides critical evidence for developing targeted psychosocial interventions.

Synthesizing the above evidence, this study proposes a hypothesized pathway: IBD-related disability may be associated with lower levels of PTG indirectly, first by being associated with lower engagement in health-promoting lifestyle (HPL), and subsequently, lower HPL may be associated with higher perceived stigma, which could be further associated with lower levels of PTG. This conceptual model aligns with contemporary psychosocial research on chronic illness. While previous studies in IBD have established mediating roles, such as quality of life between illness perception and PTG, the specific chain-mediating pathway involving HPL and stigma in the relationship between disability and PTG remains unexamined, constituting a significant gap in the current literature (16). Accordingly, this study aims to explore this potential chain mediation pathway among patients with IBD, in order to clarify the underlying psychosocial mechanisms. The findings are expected to provide theoretical support and practical references for developing targeted psychological and behavioral intervention programs.

## Methods

### Participants

Patients diagnosed with IBD were recruited from the gastroenterology department of a tertiary-level general hospital in Shanghai between August 2022 and April 2023, using a convenience sampling method. Inclusion criteria were: (1) aged 18 years or older; (2) diagnosed with IBD according to established clinical guidelines (30, 31); (3) being conscious, possessing adequate comprehension and writing abilities, and able to communicate effectively; (4) providing written informed consent. Exclusion criteria included: (1) a documented history of psychiatric disorders; (2) comorbid severe systemic diseases (e.g., malignant tumors, stroke, or heart failure); and (3) concurrent participation in other clinical trials.

All questionnaires were examined for completeness on the spot after collection. Strict quality control was implemented for missing data in this study: questionnaires with missing items or incomplete information were directly excluded and eliminated. No obvious missing data existed in the final valid 311 samples; therefore, no missing data imputation, deletion, or other supplementary statistical treatments were performed in subsequent statistical analyses. The sample size was determined using GPower 3.1 software (32). Based on an F-test for linear multiple regression, with the following parameters: significance level (*α*) = 0.05, statistical power (1-*β*) = 0.80, effect size (f^2^) = 0.15, number of tested predictors = 12, and number of groups = 1, a minimum of 172 participants was required. To accommodate potential invalid responses and increase the robustness of the chain mediation analysis, the target sample size was increased to 320. This adjustment ensures sufficient power (≥80%) for detecting both direct and indirect effects in the proposed multivariate mediation model (PROCESS Model 6), aligning with sample size estimation practices in similar observational studies of psychosocial mechanisms in IBD.

This study was conducted in accordance with the principles of the Declaration of Helsinki. All participants provided written informed consent prior to enrollment. Ethical approval was obtained from the Ethics Review Committee of Shanghai Tenth People’s Hospital (Approval No.: SHYS-IEC-5.0/22K233/P01).

### Measures

#### General information questionnaire

A self-designed questionnaire was used to collect sociodemographic and clinical characteristics. Information gathered included gender, age, marital status, educational level, monthly per capita household income, IBD subtype (UC or CD), disease duration, and current disease activity status.

#### Disease activity assessment scales

Disease activity in patients with IBD was professionally assessed in this study. Patients with CD were evaluated using the Harvey–Bradshaw Index (HBI) (33), while those with UC were assessed with the Modified Mayo Score (34). Based on the evaluation results, specialist physicians classified patients into a remission period and an active period. For CD patients, an HBI score ≤ 4 was defined as the remission period; a score of 5–8 was defined as moderate activity, and ≥9 as severe activity. For UC patients, a Modified Mayo Score ≤ 2 (with no subscore >1) was defined as the remission period; a score of 3–5 was defined as mild activity, 6–10 as moderate activity, and 11–12 as severe activity.

#### Health-promoting lifestyle profile II (HPLP-II)

Health-promoting lifestyle was assessed using the Chinese version of the Health-Promoting Lifestyle Profile II (HPLP-II) (21, 35). The original scale, developed by Pender et al., was cross-culturally adapted and validated for the Chinese population. It comprises 52 items categorized into six subscales: Health Responsibility (9 items), Physical Activity (8 items), Nutrition (9 items), Spiritual Growth (or Self-Actualization) (9 items), Interpersonal Relations (9 items), and Stress Management (8 items). Each item is rated on a 4-point Likert scale ranging from 1 (never) to 4 (always). The total score ranges from 52 to 208, with higher scores indicating a more positive health-promoting lifestyle. According to the original scoring guidelines, total scores can be categorized as follows: 52–90 (Poor), 91–129 (Fair), 130–168 (Good), and 169–208 (Excellent). In the original validation study, the overall Cronbach’s *α* coefficient was 0.93, with subscale α values ranging from 0.69 to 0.90. In the present study, the overall scale demonstrated good internal consistency, with a Cronbach’s α of 0.813.

#### Inflammatory bowel disease disability index (IBD-DI)

Disability was assessed using the Chinese version of the Inflammatory Bowel Disease Disability Index (IBD-DI) (33, 36). The original scale was developed by Peyrin-Biroulet et al., and the Chinese version was cross-culturally adapted and screened to 14 items, covering three domains: emotional function, social function, and symptom dimension. Items 1 to 13 adopt a 4-point Likert scale scored from 1 (no difficulty) to 4 (severe difficulty). Item 14 evaluates the frequency of loose stools, with a separate scoring standard: 0 times = 0 points, 1–7 times = 1 point, 8–18 times = 2 points, 19–29 times = 3 points, and ≥30 times = 4 points. The total score was calculated using the Coster-Rousseau formula: (Raw total score × 100) / (Number of completed items × 4). The standardized score ranges from 0 to 100, with higher scores indicating a higher degree of disability. Disability severity was classified as 0–12 (no disability), 13–22 (mild), 23–30 (moderate), and 31–100 (severe). The original Chinese version reported a Cronbach’s *α* of 0.866 and a split-half reliability of 0.786. In this study, the Cronbach’s α was 0.822.

#### Stigma scale for chronic illness (SSCI)

Illness-related stigma was measured using the Chinese version of the Stigma Scale for Chronic Illness (SSCI) (37, 38). The original scale was developed by Rao et al. and subsequently adapted for Chinese populations. The scale contains 24 items that form two subscales: internalized stigma (13 items) and enacted stigma (11 items). Responses are recorded on a 5-point Likert scale. The total score ranges from 24 to 120, with higher scores reflecting a higher level of perceived stigma. The Chinese version has shown good psychometric properties, with reported Cronbach’s *α* coefficients of 0.890 for internal consistency and a content validity index of 0.836. In this study, the overall Cronbach’s α for the scale was 0.816, indicating acceptable internal consistency.

#### Posttraumatic growth inventory (PTGI)

Posttraumatic growth was measured using the Chinese version of the Posttraumatic Growth Inventory (PTGI) (39, 40). The original 21-item scale developed by Tedeschi and Calhoun was cross-culturally adapted and culturally revised into a 20-item Chinese version with good psychometric characteristics for Chinese populations. The scale evaluates five domains of positive psychological changes following adversity: Relating to Others (6 items), New Possibilities (3 items), Personal Strength (4 items), Spiritual Change (3 items), and Appreciation of Life (4 items). Participants rated the degree of each perceived change on a 6-point Likert scale from 0 (I did not experience this change as a result of my crisis) to 5 (I experienced this change to a very great degree as a result of my crisis). The total score ranges from 0 to 100, with higher scores representing a higher level of posttraumatic growth. In this study, the Chinese PTGI exhibited excellent internal consistency, with a Cronbach’s *α* coefficient of 0.968.

### Survey methodology

Data were collected via anonymous, self-administered paper questionnaires. Prior to enrollment, all eligible patients received a detailed explanation of the study’s purpose, procedures, potential risks and benefits, and written informed consent was obtained from each participant. Questionnaires were distributed on-site in the outpatient clinic. The research staff was available to provide clarification if participants had any questions. For participants with reading or writing difficulties, the investigator read the questions aloud and recorded their responses verbatim. For participants with limited reading or writing ability, researchers assisted in reading items aloud and recording answers accordingly, which may inevitably introduce potential investigator bias. Participants returned the completed questionnaires directly to the research staff, who performed an immediate visual check for completeness. Out of 320 patients invited to participate, 311 returned fully completed questionnaires, yielding a valid response rate of 97.2%.

### Data analysis

Statistical analyses were performed using SPSS 26.0 (IBM Corp., Armonk, NY, USA) and the PROCESS macro (version 4.1) for SPSS. The normality of continuous variable distributions was assessed using the Shapiro–Wilk test. Continuous variables that were approximately normally distributed are presented as mean ± standard deviation (SD). Differences in scale scores across different demographic and clinical subgroups were examined using independent-samples *t*-tests (for two-group comparisons) and one-way analysis of variance (ANOVA, for comparisons of three or more groups). For the purpose of cross-scale comparability, standardized score rates were calculated for each primary scale using the formula: (Actual Total Score/Maximum Possible Total Score) × 100%. Bivariate relationships among the primary study variables (IBD-DI, HPLP-II, SSCI, and PTGI) were assessed using Pearson’s correlation coefficients.

To test the hypothesized chain mediation model, we followed the procedures outlined by Hayes (41). Disease activity and disability severity were adjusted as covariates in all mediation models to control for potential confounding effects. First, to control for potential confounding effects, demographic and clinical variables with significant between-group differences in univariate analysis (*p* < 0.05) were screened and incorporated as covariates in subsequent regression models. The hypothesized chain mediation pathway among IBD-DI, HPLP-II, SSCI and PTGI was tested using Model 6 of the PROCESS macro, with 5,000 bias-corrected bootstrap samples to estimate the indirect effects and their 95% confidence intervals (*CI*s). The indirect effects were considered statistically significant if the bootstrap 95% *CI*s did not include zero (41). In all analyses, a two-tailed *p*-value of less than 0.05 was considered statistically significant (42).

## Results

### General characteristics of patients with inflammatory bowel disease

A total of 311 patients with IBD were enrolled, with a mean age of 36.82 years, including 160 males (51.4%) and 151 females (48.6%). Most had a college/bachelor’s degree and middle-to-high household income; Crohn’s disease (54.7%) was predominant, nearly half had a disease duration of 1–5 years, and active disease (51.1%) and remission (48.9%) were nearly evenly distributed. Univariate analysis demonstrated that only disease activity and disability severity were associated with the main research indicators, which were further incorporated as covariates in the subsequent mediation model. Demographic factors including gender, age, education, household income, marital status, disease duration and disease type showed no statistical differences across all study variables (all *p* > 0.05). Detailed characteristics and comparison results are shown in Table 1.

**Table 1:** Demographic, clinical characteristics of participants and comparisons of posttraumatic growth, disability index, health-promoting lifestyle, and stigma among different subgroups (*N* = 311).

Variable	Category	Frequency (*n*)	Percentage (%)	PTG (Mean ± SD)	*Statistic*	*p*	IBD-DI (Mean ± SD)	*t/F*	*p*	HPL (Mean ± SD)	Statistic	*p*	Stigma (Mean ± SD)	Statistic	*p*
Gender	Male	160	51.4	73.52 ± 25.41	*t* = 1.897	0.059	27.02 ± 12.95	*t* = −1.396	0.164	146.91 ± 25.87	*t* = −0.814	0.416	68.25 ± 19.31	*t* = −0.914	0.361
	Female	151	48.6	68.00 ± 25.89			29.08 ± 13.02			150.32 ± 31.81			70.94 ± 22.39		
Age (years)	21 ~ 30	136	43.7	73.15 ± 25.51	*F* = 1.575	0.208	26.89 ± 12.76	*F* = 1.722	0.179	152.34 ± 28.45	*F* = 2.531	0.081	68.72 ± 29.15	*F* = 0.149	0.862
	31 ~ 40	93	29.9	70.02 ± 18.72			28.56 ± 9.01			147.89 ± 16.92			69.34 ± 23.37		
	>40	82	26.4	67.58 ± 24.68			30.12 ± 15.24			144.12 ± 27.28			70.15 ± 19.54		
Education	Junior High School and Below	31	10.0	68.51 ± 21.42	*F* = 0.598	0.619	30.25 ± 13.56	*F* = 1.420	0.237	142.36 ± 27.58	*F* = 1.179	0.318	72.45 ± 21.87	*F* = 0.702	0.550
	Senior High School/Vocational School	89	28.6	69.76 ± 15.58			28.79 ± 9.12			146.78 ± 26.89			70.12 ± 24.45		
	College/Bachelor’s Degree	140	45.0	70.88 ± 23.76			27.98 ± 12.87			149.56 ± 29.65			68.95 ± 22.21		
	Master’s degree and above	51	16.4	71.29 ± 16.03			27.56 ± 8.73			153.21 ± 23.38			65.52 ± 19.18		
*Per Capita* Monthly Household Income (RMB)	<5,000	73	23.5	66.89 ± 19.52	*F* = 1.667	0.190	30.59 ± 10.05	*F* = 2.228	0.109	146.67 ± 26.82	*F* = 1.513	0.221	74.78 ± 19.35	*F* = 2.866	0.058
	5,000–10,000	98	31.5	69.05 ± 25.61			29.42 ± 8.98			145.23 ± 19.75			69.56 ± 23.27		
	>10,000	140	45.0	72.95 ± 23.54			27.37 ± 12.92			150.95 ± 29.80			66.34 ± 21.21		
Marital status	Married	193	62.1	69.94 ± 21.53	*t* = −0.377	0.706	28.45 ± 12.96	*t* = 0.042	0.966	148.57 ± 36.78	*t* = −0.122	0.903	68.42 ± 15.28	*t* = −0.840	0.401
	Unmarried	118	37.9	71.04 ± 25.58			28.39 ± 12.91			149.03 ± 25.82			70.57 ± 23.33		
Disease duration	<1 year	85	27.3	69.88 ± 12.71	*F* = 1.763	0.155	27.95 ± 8.84	*F* = 0.335	0.800	149.67 ± 26.65	*F* = 0.487	0.691	66.95 ± 20.24	*F* = 1.392	0.245
	1 ~ <5 years	143	46.0	71.35 ± 22.42			28.47 ± 14.96			148.95 ± 19.79			69.42 ± 19.31		
	5 ~ <10 years	70	22.5	68.91 ± 19.66			28.79 ± 11.05			147.89 ± 36.92			70.15 ± 21.43		
	≥10 years	13	4.2	73.42 ± 22.19			29.12 ± 15.18			146.53 ± 31.08			71.15 ± 25.43		
Disease type	Crohn’s disease (CD)	170	54.7	71.32 ± 25.74	*t* = 1.062	0.280	29.15 ± 10.12	*t* = 1.268	0.206	150.26 ± 27.05	*t* = 1.021	0.308	71.45 ± 22.61	*t* = 1.449	0.148
	Ulcerative colitis (UC)	141	45.3	68.51 ± 21.48			27.54 ± 12.81			146.53 ± 36.62			66.72 ± 23.98		
Disease activity	Remission period	152	48.9	74.59 ± 22.32	*t* = 3.082	**0.002** ^*^	25.42 ± 7.57	*t* = −4.404	**<0.001** ^**^	151.32 ± 36.54	*t* = 1.510	0.132	67.28 ± 19.05	*t* = 1.572	0.117
	Active period	159	51.1	66.32 ± 24.87			30.87 ± 13.34			145.86 ± 24.12			71.36 ± 23.58		
Disability severity	No disability	114	36.6	75.72 ± 19.12	*F* = 5.203	**0.002** ^*^	22.36 ± 10.89	*F* = 19.083	**<0.001** ^**^	156.78 ± 35.42	*F* = 7.679	**<0.001** ^**^	65.42 ± 18.76	*F* = 2.392	0.068
	Mild disability	91	29.3	71.05 ± 23.38			28.58 ± 12.15			149.85 ± 26.71			68.95 ± 22.13		
	Moderate disability	89	28.6	66.21 ± 26.59			33.14 ± 13.68			140.56 ± 24.25			72.63 ± 21.47		
	Severe disability	17	5.5	58.16 ± 29.37			38.79 ± 7.23			129.45 ± 18.11			75.89 ± 13.02		

PTG, Posttraumatic Growth; IBD-DI, IBD Disability Index; HPL, Health-Promoting Lifestyle. Bold *p*-values indicate statistically significant differences between subgroups.

**p < 0.001, ^*^p < 0.05.

### Levels of posttraumatic growth, disability, health-promoting lifestyle, and illness-related stigma

The overall mean scores of PTGI, IBD-DI, HPLP-II and SSCI among participants were 70.84 ± 25.65, 28.39 ± 12.98, 148.61 ± 36.84 and 69.24 ± 29.33, respectively, and the subscale dimensional scores of each scale are listed in Table 2. In this cohort, 63.4% of patients had varying degrees of IBD-related disability, including mild (29.3%), moderate (28.6%) and severe disability (5.5%), while 36.6% presented no disability. Stratified analyses showed that patients in the active stage had lower PTG scores (*p* < 0.05) and higher IBD-DI scores (*p* < 0.001). Increased disability severity was accompanied by reduced PTG and HPLP-II levels, together with elevated illness-related stigma perception. No obvious differences in the four core variables were observed between CD and UC patients.

**Table 2:** Total scores and dimension scores of posttraumatic growth, disability index, health-promoting lifestyle, and stigma among patients with IBD (*n* = 311; Score, Mean ± SD; %).

Items	Number of items	Total score	Mean item score	Standardized score rate/%
Total score of health-promoting lifestyle	52	148.61 ± 36.84	2.86 ± 0.71	71.5
Interpersonal relationships	9	28.14 ± 6.53	3.13 ± 0.73	78.3
Health responsibility	9	26.94 ± 6.44	2.99 ± 0.72	74.8
Stress management	8	23.02 ± 6.60	2.88 ± 0.83	72.0
Nutrition	9	30.90 ± 3.54	3.43 ± 0.40	85.8
Physical activity	8	26.76 ± 6.50	3.34 ± 0.81	83.5
Self-actualization	9	23.93 ± 5.84	2.66 ± 0.65	66.5
Total score of disability index	14	28.39 ± 12.98	2.03 ± 0.93	50.8
Total score of stigma	24	69.24 ± 29.33	2.89 ± 1.22	57.8
Internal stigma	13	35.46 ± 13.94	2.73 ± 1.07	54.6
External stigma	11	28.77 ± 13.59	2.62 ± 1.24	52.4
Total score of posttraumatic growth	20	70.84 ± 25.65	3.54 ± 1.32	70.8
Appreciation of life	4	9.54 ± 3.46	2.37 ± 1.15	47.4
Relationships with others	6	22.80 ± 8.84	3.83 ± 1.12	76.6
Personal strength	4	12.56 ± 5.00	3.14 ± 1.25	62.8
New possibilities	3	12.85 ± 6.04	3.86 ± 1.10	77.2
Spiritual change	3	13.09 ± 2.63	3.59 ± 1.20	71.8

### Correlation analysis among posttraumatic growth, disability index, health-promoting lifestyle and illness-related stigma in IBD patients

Pearson correlation analysis showed that the disability index was negatively associated with HPLP-II (*r* = −0.711, *p* < 0.001) and PTG (*r* = −0.396, *p* < 0.001), and positively associated with illness-related stigma (*r* = 0.672, *p* < 0.001). Illness-related stigma was negatively associated with HPLP-II (*r* = −0.821, *p* < 0.001) and PTG (*r* = −0.528, *p* < 0.001). A positive association was found between HPLP-II and PTG (*r* = 0.558, *p* < 0.001). Detailed results are presented in Table 3.

**Table 3:** Correlation analysis results of posttraumatic growth, health-promoting lifestyle, stigma, and disability index among patients with IBD (*n* = 311; *r*).

Variables	Health-promoting lifestyle	Disability index	Stigma	Posttraumatic growth
Health-promoting lifestyle	1.000			
Disability index	−0.711^**^	1.000		
Stigma	−0.821^**^	0.672^**^	1.000	
Posttraumatic growth	0.558^**^	−0.396^**^	−0.528^**^	1.000

^**^p < 0.001.

### The mediating effects of health-promoting lifestyle and illness-related stigma on disability index and posttraumatic growth among patients with inflammatory bowel disease

#### Assessment of common method Bias

Common method bias was assessed using Harman’s single-factor test. Nine factors with eigenvalues greater than 1 were extracted, which together accounted for 68.34% of the total variance. The first factor explained 30.00% of the variance, which is below the 40% threshold, indicating that common method bias was not a major concern in this study. Although Harman’s single-factor test indicated no severe common method bias, subtle reporting bias caused by self-reported questionnaires could not be completely excluded.

#### Regression analysis for the chain mediation model

**Figure 1:**
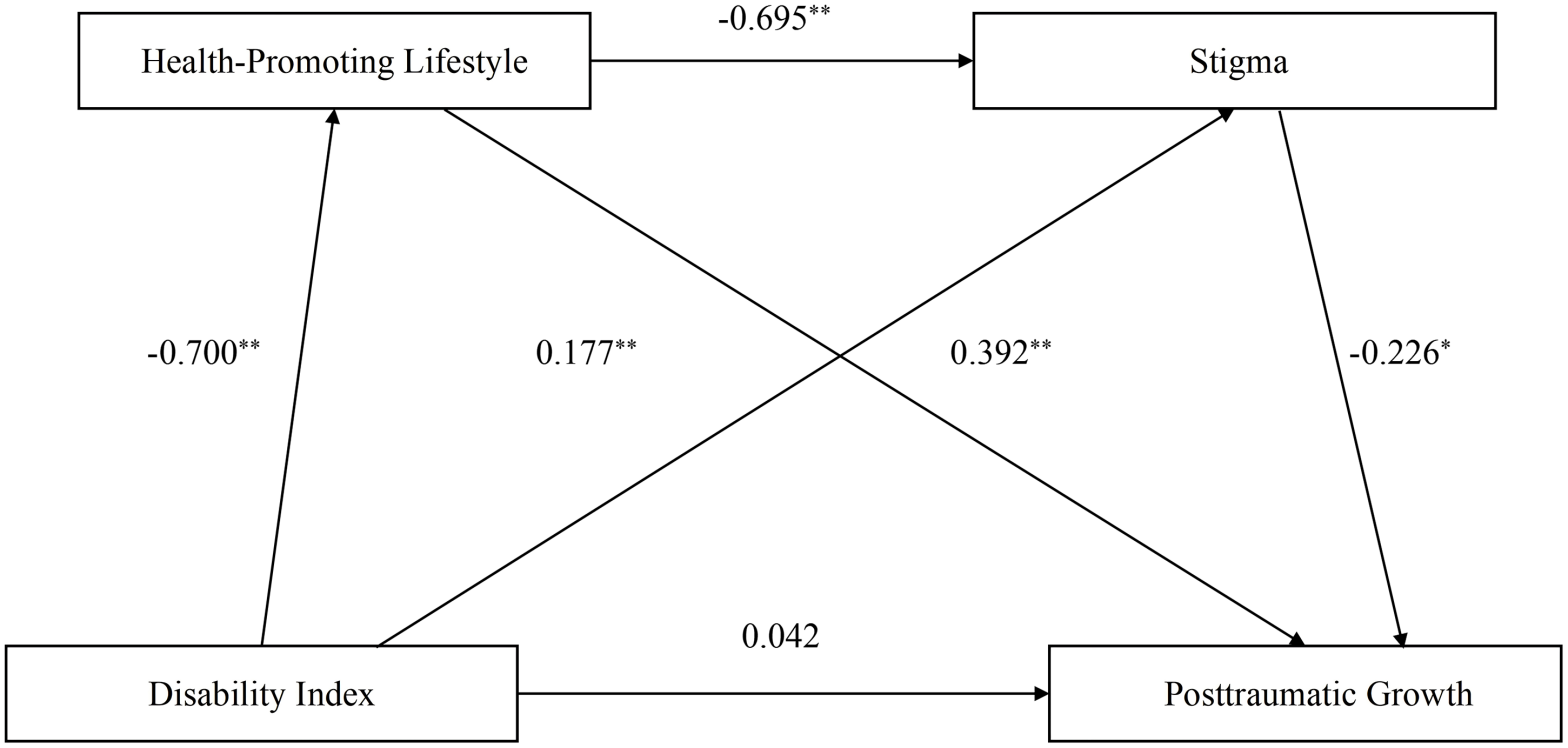
Chain mediation model of health-promoting lifestyle and stigma between disability index and posttraumatic growth. Values represent the corresponding standardized path coefficients; **p < 0.001, *p < 0.05.

All variables were standardized for the analysis. Utilizing the SPSS macro Process Model 6, a chain mediation model was tested with disability index as the independent variable, PTG as the dependent variable, and HPLP-II and illness-related stigma as chain mediators. The results of the regression analysis are presented in Table 4 and a path diagram is shown in Figure 1. To assess multicollinearity, variance inflation factors (VIF) and tolerance values were examined. All VIF values ranged from 1.014 to 3.620, which were far below the critical cutoff of 5, indicating no obvious multicollinearity among variables. The disability index was negatively associated with HPLP-II (*β* = −0.700, *p* < 0.001) and positively associated with illness-related stigma (*β* = 0.177, *p* < 0.001). HPLP-II was negatively associated with illness-related stigma (*β* = −0.695, *p* < 0.001). HPLP-II was positively associated with PTG (*β* = 0.392, *p* < 0.001), whereas illness-related stigma was negatively associated with PTG (*β* = −0.226, *p* < 0.05). The direct association between the disability index and PTG was not significant (*β* = 0.042, *p* > 0.05). Additionally, disease activity was negatively associated with HPLP-II (*β* = −0.091, *p* < 0.05) but not significantly associated with illness-related stigma or PTG (all *p* > 0.05). Disability severity was not significantly associated with HPLP-II, illness-related stigma, or PTG (all *p* > 0.05). The overall model accounted for 33.2% of the variance in PTG (*R*^2^ = 0.332), with a significant F-statistic (*F* = 30.250, *p* < 0.001), indicating a good model fit. The R^2^ values for HPLP-II and illness-related stigma regression models were 0.515 (*F* = 108.624, *p* < 0.001) and 0.690 (*F* = 170.524, *p* < 0.001), respectively.

**Table 4:** Regression analysis of chain mediating model of health-promoting lifestyle and stigma between disability index and posttraumatic growth in patients with IBD (*n* = 311).

Items	Health-promoting lifestyle			Stigma			Posttraumatic growth		
	*β*	*t*	*p*	*β*	*t*	*p*	*β*	*t*	*p*
Disability index	−0.700	−17.489	<0.001	0.177	3.914	<0.001	0.042	0.615	0.539
Health-promoting lifestyle	—	—	—	−0.695	−15.210	<0.001	0.392	4.406	<0.001
Stigma	—	—	—	—	—	—	−0.226	−2.684	0.008
Disease activity	−0.091	−2.278	0.023	0.007	0.209	0.835	−0.046	−0.965	0.335
Disability severity	−0.021	−0.534	0.593	−0.007	−0.224	0.823	−0.039	−0.823	0.411
*R^2^*	0.515			0.690			0.332		
*F*	108.624		<0.001	170.524		<0.001	30.250		<0.001
*Tolerance*	0.986			0.485			0.276		
*VIF*	1.014			2.061			3.620		

β denotes the standardized regression coefficient. Tolerance and VIF values are from the collinearity diagnostics for each predictor variable in the respective regression equation.

### Examining the chain mediation effect of health-promoting lifestyle and illness-related stigma on the association between disability index and posttraumatic growth in patients with inflammatory bowel disease

Bootstrap analysis with 5,000 resamples was used to estimate 95% confidence intervals (*CI*s) for unstandardized path coefficients, while standardized regression coefficients (*β*) are reported in Table 4. The direct effect of the disability index on PTG was not significant (unstandardized effect = 0.101, 95% *CI*: −0.222 to 0.423). The total indirect effect was significant (effect = −1.019, 95% *CI*: −1.313 to −0.755). Indirect Effect 1 (Disability → HPLP-II → PTG) showed a significant indirect effect of −0.660 (95% *CI*: −0.951 ~ −0.397), accounting for 64.71% of the total indirect effect. Indirect Effect 2 (Disability → Illness-Related Stigma → PTG) had a significant indirect effect of −0.096 (95% *CI*: −0.202 ~ −0.033), representing 9.42% of the total indirect effect. Indirect Effect 3 (Disability → HPLP-II → Illness-Related Stigma → PTG) yielded a significant indirect effect of −0.264 (95% *CI*: −0.465 ~ −0.090), accounting for 25.87% of the total indirect effect. Detailed bootstrap results are presented in Table 5.

**Table 5:** Test of chain mediation effect of health-promoting lifestyle and stigma between disability index and posttraumatic growth in patients with inflammatory bowel disease.

Items	Effect value	SE	Bootstrap 95% CI	Proportion of relative mediation effect
Disability Index → Health-Promoting Lifestyle → Posttraumatic Growth	−0.660	0.143	−0.951 ~ −0.397	64.71
Disability Index → Stigma → Posttraumatic Growth	−0.096	0.041	−0.202 ~ −0.033	9.42
Disability Index → Health-Promoting Lifestyle → Stigma → Posttraumatic Growth	−0.264	0.094	−0.465 ~ −0.090	25.87
Direct effect	0.101	0.164	−0.222 ~ 0.423	—
Indirect effect	−1.019	0.140	−1.313 ~ −0.755	100.00
Total effect	−0.918	—	—	—

## Discussion

### Current levels of health-promoting lifestyle, illness-related stigma, disability, and posttraumatic growth in IBD patients

This study reported a mean IBD disability index of 28.39 ± 12.98 and a disability prevalence of 63.4%, reflecting prominent functional impairment among included patients, which aligns with prior evidence associating chronic inflammation and sustained disease activity with daily functional limitations (9). Despite considerable IBD-related disability, participants showed moderate-to-high health-promoting behaviors and posttraumatic growth, consistent with prior studies confirming positive psychological adaptation among patients living with IBD (16). In contrast, adaptive health-promoting behaviors corresponded to individual behavioral characteristics in the IBD population (43). Meanwhile, participants reported elevated illness-related stigma, as IBD-related functional restrictions and unpredictable gastrointestinal symptoms including diarrhea and anal fistula are correlated with poor social participation and are linked to negative self-perception, which is consistent with previous evidence (44). In addition, illness-related stigma is closely associated with negative illness perceptions, and such cognitive factors are key correlates of posttraumatic growth outcomes (16). Subgroup analyses showed disparate correlations between clinical indicators and research variables. Disease activity was significantly correlated with PTG and IBD-DI scores, while health-promoting lifestyle and stigma presented closer correlation with cumulative disability severity rather than current inflammatory activity status. Clinical remission status fails to correspond to improved health behaviors or lower stigma level. As stated in STRIDE-II guidelines, disability prevention is regarded as a long-term therapeutic indicator independent from disease remission (45). In summary, IBD patients in this study exhibited high levels of disability and illness-related stigma, along with moderate health-promoting behaviors and posttraumatic growth. These descriptive findings reflect the current status of the study population and can inform individualized health guidance and psychological care in clinical practice (46).

### Health-promoting lifestyle as a mediator between disability and posttraumatic growth

The results verified a significant independent mediating role of health-promoting lifestyle on the association between disability and PTG. This pathway represented the strongest indirect effect (−0.660, accounting for 64.71% of the total indirect effect). Higher disability was correlated with reduced engagement in health-promoting behaviors, which was further associated with lower PTG. This aligns with prior research in Crohn’s disease, where better health-promoting behaviors were associated with improved disease-specific quality of life (47). Functional limitations linked to disability are closely correlated with reduced engagement in structured health behaviors, and psychosocial lifestyle factors are similarly associated with perceived disability in IBD (48). Sustained health-promoting behaviors represent a positive psychological adaptation among individuals with chronic illness. Existing studies demonstrate that PTG in young IBD patients is closely correlated with restored life autonomy and cognitive reframing under long-term disease burden (49). Adherence to a healthy lifestyle reflects positive psychological adjustment in disease management, which may act as a vital mental resource for patients with significant disability. Similarly, studies focusing on Chinese IBD populations have indicated that positive mindset is closely associated with improved coping and long-term adaptation to illness-related stress and physical challenges (50). Regular healthy behaviors are correlated with milder clinical symptoms and restored life control, which links to lower disability-related distress and higher PTG. Interventions including narrative health education and online peer support are associated with better health behavioral adherence and long-term quality of life among IBD patients (46). The prominent mediating role of health-promoting lifestyle indicates that IBD clinical management can extend beyond routine disease education to integrated, behavior-oriented support. Future care frameworks may combine standardized lifestyle guidance with disease self-management, which correlates with favorable psychological outcomes and adaptive posttraumatic growth amid chronic disability. The strong negative correlation (*r* = −0.821) between health-promoting lifestyle and stigma indicates a clinically relevant cyclic association, rather than statistical multicollinearity. Disability status is correlated with compromised healthy behaviors and elevated stigma perception, while higher stigma level coexists with less favorable health behavioral performance. This pathway occupies 64.71% of the total indirect effect, suggesting lifestyle intervention serves as a promising strategy to mitigate such unfavorable linkage. Both factors are independently associated with quality of life apart from posttraumatic growth, and this interactive correlation has been documented in recent literature (51).

### Illness-related stigma exerts a unique mediating effect between the IBD disability index and posttraumatic growth

This study confirmed the mediating role of illness-related stigma, with a significant yet relatively limited indirect effect (−0.096, accounting for 9.42%). Although its effect size was markedly smaller than that of the HPLP-II pathway, this finding identifies illness-related stigma as an independent pathway correlated with psychological adaptation among individuals with disability (44, 48). Higher disability is correlated with a negative self-perception of incapability, which presents parallel trends with internalized illness-related stigma and coincides with reduced psychological resource status (26). Notably, the connection between illness-related stigma and psychological outcomes is not uniformly negative. Psychological distress correlated with illness-related stigma may, in certain contexts, align with cognitive processes related to meaning-making and reappraisal (52). In this study, lower illness-related stigma levels, together with active health-promoting behaviors, were correlated with adaptive psychological status and higher PTG. Interventions including narrative-based health education and targeted anti-stigma programs are associated with positive illness cognition and personal psychological perception, showing parallel trends with the psychological dimensions linked to illness-related stigma (46). Accordingly, illness-related stigma constitutes a vital psychological pathway in the correlation between disability and PTG. Clinical care protocols may combine illness-related stigma regulation and psychological adaptation training with routine symptom management, to support holistic psychological well-being among patients experiencing chronic IBD stress.

### Health-promoting lifestyle and illness-related stigma mediate the relationship between IBD disability index and posttraumatic growth

A primary contribution of this study is the verification of a significant chain mediation pathway involving HPLP-II and illness-related stigma (indirect effect = −0.264, 25.87% of the total indirect effect). This pathway holds theoretical integrative value, revealing that the two mediators do not operate in isolation: disability is first correlated with reduced HPLP-II engagement, and diminished HPLP-II is associated with weakened positive self-image, which in turn correlates with increased internalized illness-related stigma, forming a sequential negative association from behavior to cognition (53). First, the physical and psychological burdens of significant disability are closely associated with barriers to maintaining structured health behaviors (51). Second, a pattern of less healthy living may be associated with a worsening self-image and increased internalized illness-related stigma, as it aligns with a patient’s self-identification as “ill” or “incapable” (28, 51). From the perspective of self-regulation and social cognitive theory, insufficient engagement in positive health behaviors may exacerbate negative self-evaluation, amplify the self-labeling of chronic illness vulnerability, and further strengthen internalized stigmatized cognition, which provides tentative theoretical rationality for the correlation between decreased HPLP-II and increased illness-related stigma (54, 55). This chain mediation model revealed an indirect association between IBD disability and PTG through sequential behavioral and psychosocial pathways. Meanwhile, the model presented adequate goodness-of-fit, low multicollinearity and stable bootstrap results, which verified the robustness of all associative pathways in this study. The total association is fully mediated via combined indirect pathways, with no significant direct effect identified (16, 46). Sustained positive lifestyle engagement is associated with better symptom control and lower complication risk, both of which are important correlates of positive psychological adaptation (56). Future longitudinal studies are required to consolidate the theoretical foundation of this sequential pathway. By elucidating the above mediating mechanisms, this study offers modifiable intervention targets for clinical practice. Subsequent research could explore relevant moderators and adopt longitudinal designs to clarify the dynamic associations between variables, so as to facilitate comprehensive recovery in patients with IBD (47).

## Limitations and future directions

This study has several limitations that should be acknowledged. First, regarding design and generalizability, the cross-sectional data restricts insights into the temporal dynamics and sequential order of observed relationships. Accordingly, temporal sequences and causal inferences among variables cannot be established in the present cross-sectional design. Additionally, the convenience sample from Shanghai tertiary hospitals may limit the findings’ applicability to the broader Chinese IBD population, such as rural residents or those receiving primary care. Second, regarding measurement methods, all data were collected via self-reported questionnaires, which are susceptible to social desirability and recall biases. Although researchers provided brief clarifications to aid comprehension, such guidance may introduce slight measurement bias; incorporating objective clinical metrics in future studies will enhance findings’ validity. Third, methodological and statistical considerations require attention: (1) Common Method Variance: Harman’s single-factor test showed no dominant common factor, but the test’s inherent limitations mean potential common method bias cannot be fully ruled out. (2) Interrelated Constructs: The two mediators, HPLP-II and illness-related stigma, exhibited a strong correlation (*r* = −0.821). The strong correlation between the two mediators may imply conceptual overlap, which should be interpreted cautiously in pathway differentiation. Although collinearity diagnostics (VIF: 1.014–3.620) verified stable regression results, the strong correlation reflected close conceptual overlap between the two mediators, which may hinder the accurate differentiation of their independent roles in the chain mediation model. (3) Sample size limitation: The initial sample size calculation relied on conventional multiple regression F-tests. For complex chain mediation models, simulation-based methods such as Monte Carlo analysis can support more rigorous and precise sample size planning.

For future research, several directions are proposed. Methodologically, longitudinal studies are needed to clarify the temporal sequence of the observed associations. Multi-center studies encompassing diverse geographic and healthcare settings would enhance the representativeness of findings. Employing more robust statistical controls for common method bias and using simulation for sample size estimation in complex models are also recommended. Substantively, future investigations could examine the roles of additional psychological and contextual variables as potential moderators or complementary mediators. Exploring how disease-specific characteristics interact with psychosocial variables may provide a more integrated understanding. Ultimately, developing and evaluating interventions focused on the identified pathways—enhancing health-promoting lifestyle and addressing illness-related stigma—could help translate these observational findings into supportive care strategies for patients with IBD.

### Implications

#### Theoretical implications

This study provides several important theoretical contributions. First, it clarifies the correlative pathways linking disability and PTG in IBD by identifying HPLP-II and illness-related stigma as sequential mediators. This addresses a gap in the literature regarding the specific correlative patterns through which functional impairment may be connected to psychological adaptation outcomes (57). Second, by integrating concepts from disability studies, health behavior models, and stigma theory, this research advances a more comprehensive understanding of the correlative network surrounding psychological adaptation in chronic illness (57). Third, the findings offer a theoretical framework for developing multi-component psychosocial interventions that target the identified correlative pathways to potentially enhance PTG in the IBD population (58).

#### Practical implications

The findings offer actionable insights for clinical practice. First, routine assessment of health-promoting behaviors and perceived illness-related stigma could be integrated into IBD care. Based on identified patterns, personalized supports—such as tailored activity guidance or approaches for illness-related stigma awareness—might be beneficial (19). Second, healthcare teams could address illness-related stigma through psychoeducation that normalizes the IBD experience and fosters self-acceptance, which may encourage patients to seek social connection (23). Third, for patients with significant functional limitations, rehabilitation supports that address specific activity challenges might facilitate greater engagement in health-promoting behaviors. Finally, a coordinated, multidisciplinary care model appears valuable. Collaboration among gastroenterologists, nurses, and psychologists can provide integrated support addressing the observed correlative patterns involving disability, health behaviors, illness-related stigma, and PTG (58).

## Conclusion

This study of 311 patients with IBD revealed substantial levels of both disability and posttraumatic growth, with greater disability correlating significantly with lower PTG. The core finding is that this association is fully mediated by the sequential pathway of HPLP-II and illness-related stigma; after accounting for these two mediators, the direct link between disability and PTG was no longer evident. These results highlight HPLP-II and illness-related stigma as critical, interconnected pathways that completely account for the relationship between disease-related disability and psychological adaptation in IBD. Accordingly, clinical and psychosocial interventions aiming to promote adaptive health behaviors and mitigate internalized illness-related stigma may serve as effective strategies to foster posttraumatic growth in this population.

## Data Availability

The raw data supporting the conclusions of this article will be made available by the authors, without undue reservation.

## References

[1] Le Berre C Honap S Peyrin-Biroulet L. Ulcerative colitis. Lancet. (2023) 402:571–84. doi: 10.1016/S0140-6736(23)00966-2 37573077

[2] Dolinger M Torres J Vermeire S. Crohn's disease. Lancet. (2024) 403:1177–91. doi: 10.1016/S0140-6736(23)02586-2, 38437854

[3] Kaplan GG Ng SC. Understanding and preventing the global increase of inflammatory bowel disease. Gastroenterology. (2017) 152:313–321.e2. doi: 10.1053/j.gastro.2016.10.020, 27793607

[4] Wan J Zhou J Wang Z Liu D Zhang H Xie S . Epidemiology, pathogenesis, diagnosis, and treatment of inflammatory bowel disease: insights from the past two years. Chin Med J. (2025) 138:763–76. doi: 10.1097/CM9.0000000000003542, 39994836 PMC11970819

[5] Bloomfeld RS Bickston SJ. Financial toxicity in people with inflammatory bowel disease. Inflamm Bowel Dis. (2021) 27:1170–1. doi: 10.1093/ibd/izaa267, 33146376

[6] The Lancet Gastroenterology & Hepatology. The economic burden of inflammatory bowel disease. Lancet Gastroenterol Hepatol. (2023) 8:391. doi: 10.1016/S2468-1253(23)00075-4, 37030297

[7] Barberio B Zamani M Black CJ Savarino EV Ford AC. Prevalence of anxiety and depression symptoms in patients with inflammatory bowel disease: a systematic review and meta-analysis. Lancet Gastroenterol Hepatol. (2021) 6:359–70. doi: 10.1016/S2468-1253(21)00014-5, 33721557

[8] Tannoury J Nachury M Martins C Serrero M Filippi J Roblin X . Determinants of IBD-related disability: a cross-sectional survey from the GETAID. Aliment Pharmacol Ther. (2021) 53:1098–107. doi: 10.1111/apt.16353, 33817819

[9] Peyrin-Biroulet L Cieza A Sandborn WJ Kostanjsek N Kamm MA Hibi T . Disability in inflammatory bowel diseases: developing ICF core sets for patients with inflammatory bowel diseases based on the international classification of functioning, disability, and health. Inflamm Bowel Dis. (2010) 16:15–22. doi: 10.1002/ibd.21010, 19610077

[10] Lo B Prosberg MV Gluud LL Chan W Leong RW van der List E . Systematic review and meta-analysis: assessment of factors affecting disability in inflammatory bowel disease and the reliability of the inflammatory bowel disease disability index. Aliment Pharmacol Ther. (2018) 47:6–15. doi: 10.1111/apt.14373 28994131

[11] Nardone OM Calabrese G Ford AC Castiglione F Savarino EV Jairath V . Prevalence of disability in inflammatory bowel disease: a systematic review and meta-analysis. Inflamm Bowel Dis. (2026):izag022. doi: 10.1093/ibd/izag022, 41784159 PMC13337221

[12] Han X Zhang XQ Chen Y Yubei G Jie L Yue L . A survey on disability status of patients with inflammatory bowel disease in China. Chin J Dig. (2023) 43:321–6. doi: 10.3760/cma.j.cn311367-20221126-00585

[13] Yu Z Ruan G Bai X Sun Y Yang H Qian J. Growing burden of inflammatory bowel disease in China: findings from the global burden of disease study 2021 and predictions to 2035. Chin Med J. (2024) 137:2851–9. doi: 10.1097/CM9.0000000000003345, 39501850 PMC11649283

[14] Chen D Zhu T Zhou Y. Constructing and preliminary testing a narrative therapy programme for posttraumatic growth in Chinese patients with inflammatory bowel disease: a pilot study. Clin Psychol Psychother. (2024) 31:e3013. doi: 10.1002/cpp.3013, 38785414

[15] Duan R Mei W Lei M Chen D Pan T Kong F . Care needs profiles of Crohn's disease patients and their associations with symptom clusters, post-traumatic growth, and family function: a latent profile analysis. BMC Gastroenterol. (2025) 25:351. doi: 10.1186/s12876-025-03953-5, 40346452 PMC12063445

[16] Hamama-Raz Y Nativ S Hamama L. Post-traumatic growth in inflammatory bowel disease patients: the role of illness cognitions and physical quality of life. J Crohns Colitis. (2021) 15:1060–7. doi: 10.1093/ecco-jcc/jjaa247, 33252614

[17] Theodoratou M Diamanti H. Understanding the traumatic impact of serious chronic illness. Eur Psychiatry. (2024) 67:S373–4. doi: 10.1192/j.eurpsy.2024.767, 40012026

[18] Ananthakrishnan AN Kaplan GG Bernstein CN Burke KE Lochhead PJ Sasson AN . Lifestyle, behaviour, and environmental modification for the management of patients with inflammatory bowel diseases: an international organization for study of inflammatory bowel diseases consensus. Lancet Gastroenterol Hepatol. (2022) 7:666–78. doi: 10.1016/S2468-1253(22)00021-8, 35487235

[19] Ho FF Gao YY Chen Y Wang BH Wu JCY Zheng H . Association of healthy lifestyle behaviours with incident inflammatory bowel disease: a population-based prospective cohort study. Aliment Pharmacol Ther. (2025) 61:1519–31. doi: 10.1111/apt.70031, 39957597

[20] Pender NJ Murdaugh CL Parsons MA. Health Promotion in Nursing Practice. Boston: Pearson (2011).

[21] Walker SN Sechrist KR Pender NJ. The health-promoting lifestyle profile: development and psychometric characteristics. Nurs Res. (1987) 36:76–81. doi: 10.1097/00006199-198703000-00002, 3644262

[22] Chen J Xiang X Lee JLC Chen C He Y Lou VWQ. Physical activity and posttraumatic growth: a systematic review of quantitative and qualitative studies. Psychol Sport Exerc. (2020) 49:101679. doi: 10.1016/j.psychsport.2020.101679

[23] Dibley L Norton C Whitehead E. The experience of stigma in inflammatory bowel disease: an interpretive (hermeneutic) phenomenological study. J Adv Nurs. (2018) 74:838–51. doi: 10.1111/jan.13492, 29105144

[24] Guo L Rohde J Farraye FA. Stigma and disclosure in patients with inflammatory bowel disease. Inflamm Bowel Dis. (2020) 26:1010–6. doi: 10.1093/ibd/izz260 32556190

[25] Taft TH Keefer L Leonhard C Nealon-Woods M. Impact of perceived stigma on inflammatory bowel disease patient outcomes. Inflamm Bowel Dis. (2009) 15:1224–32. doi: 10.1002/ibd.20864, 19180581 PMC2938734

[26] Jacks CM Stutts LA. The effect of disclosure on enacted stigma towards individuals with inflammatory bowel disease. J Clin Psychol Med Settings. (2025) 32:517–25. doi: 10.1007/s10880-025-10070-8, 40042773 PMC12370857

[27] Roberts CM Gamwell KL Baudino MN Edwards CS Jacobs NJ Tung J . Illness stigma, body image dissatisfaction, thwarted belongingness and depressive symptoms in youth with inflammatory bowel disease. Eur J Gastroenterol Hepatol. (2022) 34:919–24. doi: 10.1097/MEG.0000000000002420, 35913777

[28] Lenti MV Cococcia S Ghorayeb J Di Sabatino A Selinger CP. Stigmatisation and resilience in inflammatory bowel disease. Intern Emerg Med. (2020) 15:211–23. doi: 10.1007/s11739-019-02268-0, 31893346 PMC7054377

[29] Luo D Zhou M Sun L Lin Z Bian Q Liu M . Resilience as a mediator of the association between perceived stigma and quality of life among people with inflammatory bowel disease. Front Psych. (2021) 12:709295. doi: 10.3389/fpsyt.2021.709295, 34421685 PMC8377363

[30] Chinese Medical Association Inflammatory Bowel Disease Group. Chinese clinical practice guideline on the management of ulcerative colitis (2023, Xi'an). Chin J Inflamm Bowel Dis. (2024) 44:73–99. doi: 10.3760/cma.j.cn311367-20240125-00036

[31] Chinese Medical Association Inflammatory Bowel Disease Group. Chinese clinical practice guideline on the management of Crohn's disease (2023, Guangzhou). Chin J Inflamm Bowel Dis. (2024) 8:2–32. doi: 10.3760/cma.j.cn101480-20240108-00006

[32] Faul F Erdfelder E Lang AG Buchner A. G*power 3: a flexible statistical power analysis program for the social, behavioral, and biomedical sciences. Behav Res Methods. (2007) 39:175–91. doi: 10.3758/BF03193146, 17695343

[33] Han X Tan W. Study on reliability and validity of Chinese version of inflammatory bowel disease disability index. Chin J Inflamm Bowel Dis. (2020) 4:304–10. doi: 10.3760/cma.j.cn101480-20190910-00113

[34] Sandborn WJ Feagan BG Hanauer SB Lochs H Löfberg R Modigliani R . A review of activity indices and efficacy endpoints for clinical trials of medical therapy in adults with Crohn's disease. Gastroenterology. (2002) 122:512–30. doi: 10.1053/gast.2002.31072, 11832465

[35] Chen WJ Ge YG Pu WP Zhang JZ. Development and psychometric testing of the Chinese version of the health-promoting lifestyle profile II (HPLP-II). Chin J Dis Control Prev. (2016) 20:286–9. doi: 10.16462/j.cnki.zhjbkz.2016.03.018

[36] Peyrin-Biroulet L Cieza A Sandborn WJ Coenen M Chowers Y Hibi T . Development of the first disability index for inflammatory bowel disease based on the international classification of functioning, disability and health. Gut. (2012) 61:241–7. doi: 10.1136/gutjnl-2011-300049, 21646246 PMC3245899

[37] Rao D Choi SW Victorson D Bode R Peterman A Heinemann A . Measuring stigma across neurological conditions: the development of the stigma scale for chronic illness (SSCI). Qual Life Res. (2009) 18:585–95. doi: 10.1007/s11136-009-9475-1, 19396572 PMC2875076

[38] Dong CY Li Q Zhao Y. Development of the Chinese version of stigma scale for chronic illness and test of its reliability and validity in stroke patients. Chin Gen Pract. (2017) 20:4304–9. doi: 10.3969/j.issn.1007-9572.2017.34.018

[39] Tedeschi RG Calhoun LG. The posttraumatic growth inventory: measuring the positive legacy of trauma. J Trauma Stress. (1996) 9:455–71. doi: 10.1007/bf02103658, 8827649

[40] Ji W Wang YB Li XH Yao C. Revision of the posttraumatic growth inventory and testing its reliability and validity. J Nurs Sci. (2011) 26:26–8. doi: 10.3870/hlxzz.2011.14.026

[41] Preacher KJ Hayes AF. Asymptotic and resampling strategies for assessing and comparing indirect effects in multiple mediator models. Behav Res Methods. (2008) 40:879–91. doi: 10.3758/brm.40.3.879, 18697684

[42] Hayes AF Rockwood NJ. Regression-based statistical mediation and moderation analysis in clinical research: observations, recommendations, and implementation. Behav Res Ther. (2017) 98:39–57. doi: 10.1016/j.brat.2016.11.001, 27865431

[43] Demers K Hendrix EMB Ardabili AR Bredero QM van Bodegraven AA van der Horst D . Lifestyle and psychosocial factors in inflammatory bowel disease: prevalence, impact, motivation, and support needs. PLoS One. (2025) 20:e0331092. doi: 10.1371/journal.pone.0331092, 40880377 PMC12396644

[44] Muse K Johnson E David AL. A feeling of otherness: a qualitative research synthesis exploring the lived experiences of stigma in individuals with inflammatory bowel disease. Int J Environ Res Public Health. (2021) 18:8038. doi: 10.3390/ijerph18158038, 34360327 PMC8345596

[45] Tse CS Hunt MG Brown LA Lewis JD. Inflammatory bowel diseases-related disability: risk factors, outcomes, and interventions. Inflamm Bowel Dis. (2024) 30:501–7. doi: 10.1093/ibd/izad182, 37603844

[46] Zhang Y Pi B Xu X Li Y Chen X Yang N. Influence of narrative medicine-based health education combined with an online patient mutual assistance group on the health of patients with inflammatory bowel disease and arthritis. Psychol Res Behav Manag. (2020) 13:1–10. doi: 10.2147/PRBM.S213587, 32021504 PMC6954847

[47] Lamers CR de Roos NM Heerink HH van de Worp-Kalter LA Witteman BJM. Lower impact of disease on daily life and less fatigue in patients with inflammatory bowel disease following a lifestyle intervention. Inflamm Bowel Dis. (2022) 28:1791–9. doi: 10.1093/ibd/izac027, 35212382 PMC9713506

[48] van der Have M Fidder HH Leenders M Kaptein AA van der Valk M van Bodegraven A . Self-reported disability in patients with inflammatory bowel disease largely determined by disease activity and illness perceptions. Inflamm Bowel Dis. (2015) 21:369–77. doi: 10.1097/MIB.0000000000000278, 25569738

[49] Wu Q Zhu P Liu X Ji Q Qian M. Nirvana: a qualitative study of posttraumatic growth in adolescents and young adults with inflammatory bowel disease. Children (Basel). (2022) 9:879. doi: 10.3390/children9060879, 35740816 PMC9222066

[50] Wang Y Li S Gong J Cao L Xu D Yu Q . Perceived stigma and self-efficacy of patients with inflammatory bowel disease-related stoma in China: a cross-sectional study. Front Med. (2022) 9:813367. doi: 10.3389/fmed.2022.813367, 35252252 PMC8888524

[51] Giri S Harindranath S Chandnani S Jena A Sharma V Sebastian S. Systematic review: stigma associated with inflammatory bowel disease. Aliment Pharmacol Ther. (2025) 62:1066–88. doi: 10.1111/apt.70448 41186070

[52] Wang Y Zhang C Zhou Y. Reconstructing self from the illness: a constructivist grounded theory study of posttraumatic growth in patients with Crohn's disease. BMC Gastroenterol. (2023) 23:244. doi: 10.1186/s12876-023-02878-1, 37464276 PMC10354879

[53] Rozich JJ Holmer A Singh S. Effect of lifestyle factors on outcomes in patients with inflammatory bowel diseases. Am J Gastroenterol. (2020) 115:832–40. doi: 10.14309/ajg.0000000000000608, 32224703 PMC7274876

[54] Bandura A. Social cognitive theory of self-regulation. Organ Behav Hum Decis Process. (1991) 50:248–87. doi: 10.1016/0749-5978(91)90022-L

[55] Leventhal H Phillips LA Burns E. The common-sense model of self-regulation (CSM): a dynamic framework for understanding illness self-management. J Behav Med. (2016) 39:935–46. doi: 10.1007/s10865-016-9782-2 27515801

[56] Lopes EW Chan SSM Song M Ludvigsson JF Håkansson N Lochhead P . Lifestyle factors for the prevention of inflammatory bowel disease. Gut. (2022) 72:1093–100. doi: 10.1136/gutjnl-2022-328174, 36591609 PMC10241983

[57] Nardone OM Calabrese G La Mantia A Caso R Testa A Castiglione F. Insights into disability and psycho-social care of patients with inflammatory bowel disease. Front Med. (2024) 11:1416054. doi: 10.3389/fmed.2024.1416054, 38863889 PMC11165103

[58] Ji Q Zhang L Xu J Ji P Song M Chen Y . The relationship between stigma and quality of life in hospitalized middle-aged and elderly patients with chronic diseases: the mediating role of depression and the moderating role of psychological resilience. Front Psych. (2024) 15:1346881. doi: 10.3389/fpsyt.2024.1346881, 38840950 PMC11151782

